# Sharing genetic variants with the NGS pipeline is essential for effective genomic data sharing and reproducibility in health information exchange

**DOI:** 10.1038/s41598-021-82006-9

**Published:** 2021-01-26

**Authors:** Jeong Hoon Lee, Solbi Kweon, Yu Rang Park

**Affiliations:** 1 Lunit Inc., 175 Yeoksamro, Gangnam-gu, Seoul, Republic of Korea; 2 Department of Biomedical Systems Informatics, Yonsei University College of Medicine, Seoul, Republic of Korea

**Keywords:** Data publication and archiving, Data processing, Quality control

## Abstract

Genetic variants causing underlying pharmacogenetic and disease phenotypes have been used as the basis for clinical decision-making. However, due to the lack of standards for next-generation sequencing (NGS) pipelines, reproducing genetic variants among institutions is still difficult. The aim of this study is to show how many important variants for clinical decisions can be individually detected using different pipelines. Genetic variants were derived from 105 breast cancer patient target DNA sequences via three different variant-calling pipelines. HaplotypeCaller, Mutect2 tumor-only mode in the Genome Analysis ToolKit (GATK), and VarScan were used in variant calling from the sequence read data processed by the same NGS preprocessing tools using Variant Effect Predictor. GATK HaplotypeCaller, VarScan, and MuTect2 found 25,130, 16,972, and 4232 variants, comprising 1491, 1400, and 321 annotated variants with ClinVar significance, respectively. The average number of ClinVar significant variants in the patients was 769.43, 16.50% of the variants were detected by only one variant caller. Despite variants with significant impact on clinical decision-making, the detected variants are different for each algorithm. To utilize genetic variants in the clinical field, a strict standard for NGS pipelines is essential.

## Introduction

Genome or exome sequencing using next-generation sequencing (NGS) technologies has now entered medical practice^1^. Genetic variant databases for clinical applications were built on numerous studies of human genetic variants affecting response to medications associated with diseases and phenotypes^2–4^. As guidelines for the interpretation of sequence variants have been established, clinical laboratories now perform genetic testing for therapeutic decision-making and disease prediction. Nonetheless, the construction of uniform standards for NGS pipelines is difficult because of various genetic testing techniques, different experimental goals, and numerous algorithms^5^. As a result, clinical laboratories and medical institutions have generated patients’ genetic variants through different sequencing protocols and NGS pipelines, leading to genetic variants that are not interoperable.

The current gold standard for variant-calling pipelines is the Genome Analysis Toolkit (GATK) Best Practices Workflow pipeline using HaplotypeCaller, which is considered to have the highest accuracy for single nucleotide polymorphisms (SNPs) and small insertions and deletions^6,7^. However, the development of numerous NGS sequencing technologies, such as Illumina and BGI, has caused data-specific effects, making it difficult to build a uniform pipeline^8,9^. Data-specific effects cause false positive detection due to unexpected systematic error patterns in the HaplotypeCaller algorithm using GATK Best Practices^10^. Therefore, it is difficult to build NGS pipeline guidelines and make genetic variants interoperable in clinical practice.

The importance of reliable genetic data communication between hospitals and clinical genomic data sharing to improving genetic health care is widely recognized, and the practice has been encouraged by both professional societies and funding agencies^11^. Before sharing genetic variant data derived from raw sequencing data, the validity of the variant-calling pipeline result must be verifiable. However, different NGS pipelines among institutions produce different variant calling results despite the same raw sequencing data, causing serious problems in clinical decision-making and genetic variant sharing. Hence, diagnostic genetic tests used as a basis for clinical decision-making should be reproducible or replicable^12^.

This study suggests that the pipeline throughout the variant-calling process, including raw sequencing data, should be shared for the reproducibility of the genetic variants as a laboratory test. Of the genetic variants called by different NGS pipelines, we quantified the important variants missed, which consequently affected clinical decision-making.

## Results

**Figure 1:**
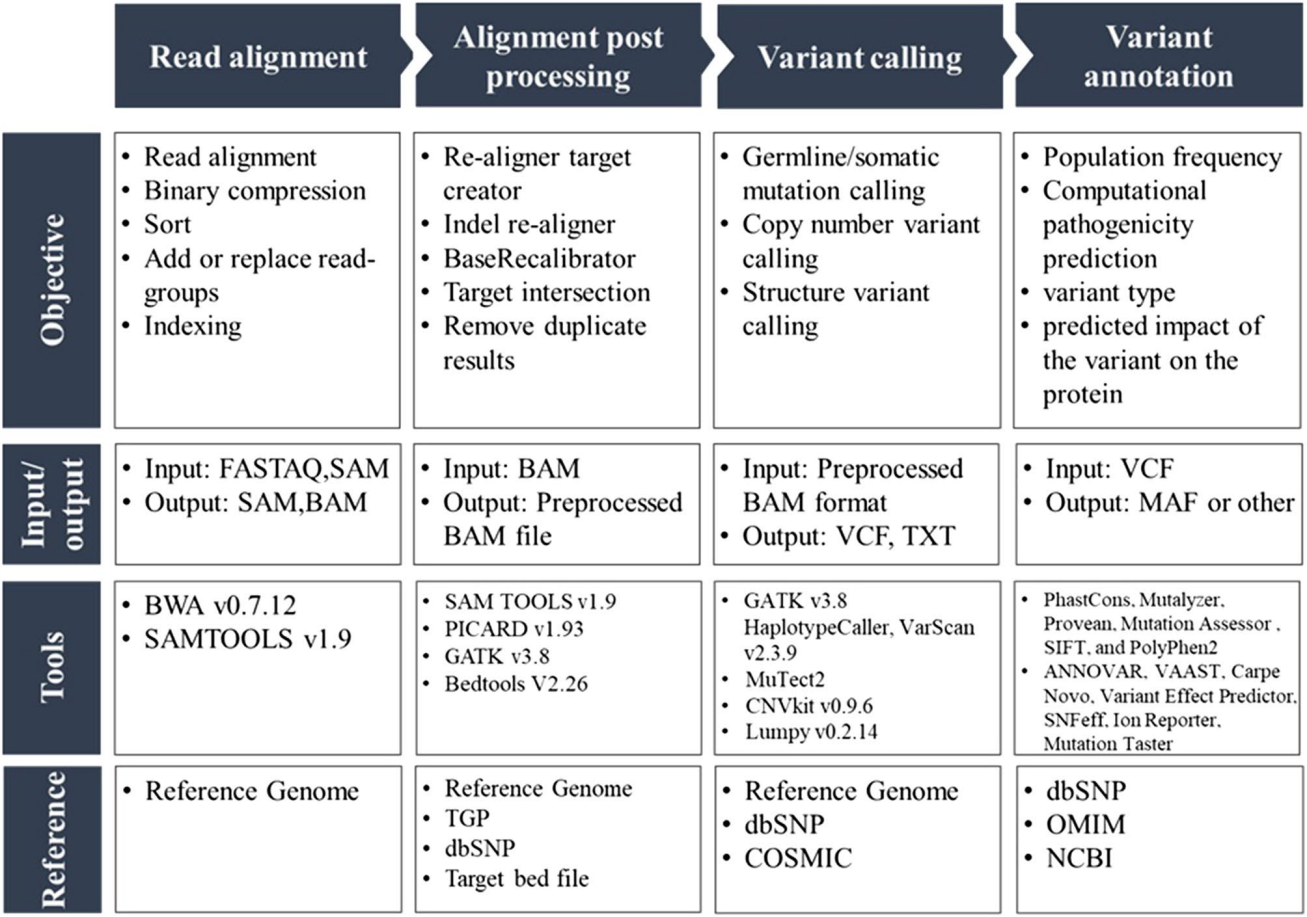
Workflow scheme for NGS preprocessing showing the program names, versions used, options, parameters, and additional files required.

Raw sequencing data were preprocessed using the GATK Best Practices-based NGS pipeline. Variant calling was performed using three different variant callers, GATK HC, VarScan, and MuTect2 tumor-only mode. Figure 1 summarizes the NGS pipeline workflow for the preprocessing of raw sequencing data. The workflow includes information about the purpose of the process, name of the program, version, options, and additional input needed for each process. The command line for all data processing is available in the supplementary data.

### The consequence of the called variants

The counts of variants called by three variant callers, HC, VarScan, and MuTect2 tumor-only mode, for aggregation of all patients are shown in Table 1. The number of called variants was highest with GATK HC, followed by VarScan and MuTect2. The average number of variants per person was 4152.362, 2925.257, and 159.219 in GATK HC, VarScan, and MuTect2, respectively. The truncation mutation, called the loss of function, is splice_acceptor_variant, splice_donor_variant, splice_region_variant, and stop_gained. The numbers of truncation mutations in GATK HC, VarScan, and MuTect2 variants were 5792 (1.33%), 4676 (1.52%), and 287 (1.72%), respectively. Based on the GATK HC, the odds ratios of the truncation mutations for all VarScan and MuTect2 variants were 1.15 and 1.29, respectively.

**Table 1:** Distribution of consequences of genetic variants using three different variant callers.

Consequence	GATK HC	Varsha	MuTect2
3_prime_UTR_variant	58,135 (13.33%)	52,305 (17.03%)	4013 (24.00%)
5_prime_UTR_variant	12,376 (2.84%)	9712 (3.16%)	444 (2.66%)
Downstream_gene_variant	42,903 (9.84%)	32,376 (10.54%)	1933 (11.56%)
Intron_variant	249,984 (57.34%)	156,050 (50.81%)	8259 (49.40%)
Missense_variant	2310 (0.53%)	2046 (0.67%)	35 (0.21%)
Non_coding_transcript_exon_variant	6349 (1.46%)	5702 (1.86%)	204 (1.22%)
Regulatory_region_variant	776 (0.18%)	681 (0.22%)	21 (0.13%)
Splice_acceptor_variant	12 (0.00%)	9 (0.00%)	0 (0.00%)
Splice_donor_variant	162 (0.04%)	63 (0.02%)	3 (0.02%)
Splice_region_variant	5302 (1.22%)	4350 (1.42%)	276 (1.65%)
Start_lost	0 (0.00%)	0 (0.00%)	1 (0.01%)
Stop_gained	316 (0.07%)	254 (0.08%)	8 (0.05%)
Stop_lost	5 (0.00%)	5 (0.00%)	1 (0.01%)
Synonymous_variant	28,813 (6.61%)	25,128 (8.18%)	467 (2.79%)
Upstream_gene_variant	28,555 (6.55%)	18,471 (6.01%)	1053 (6.30%)
Sum	435,998 (100.00%)	307,152 (100.00%)	16,718 (100.00%)

### The deleteriousness of the called variants

**Figure 2:**
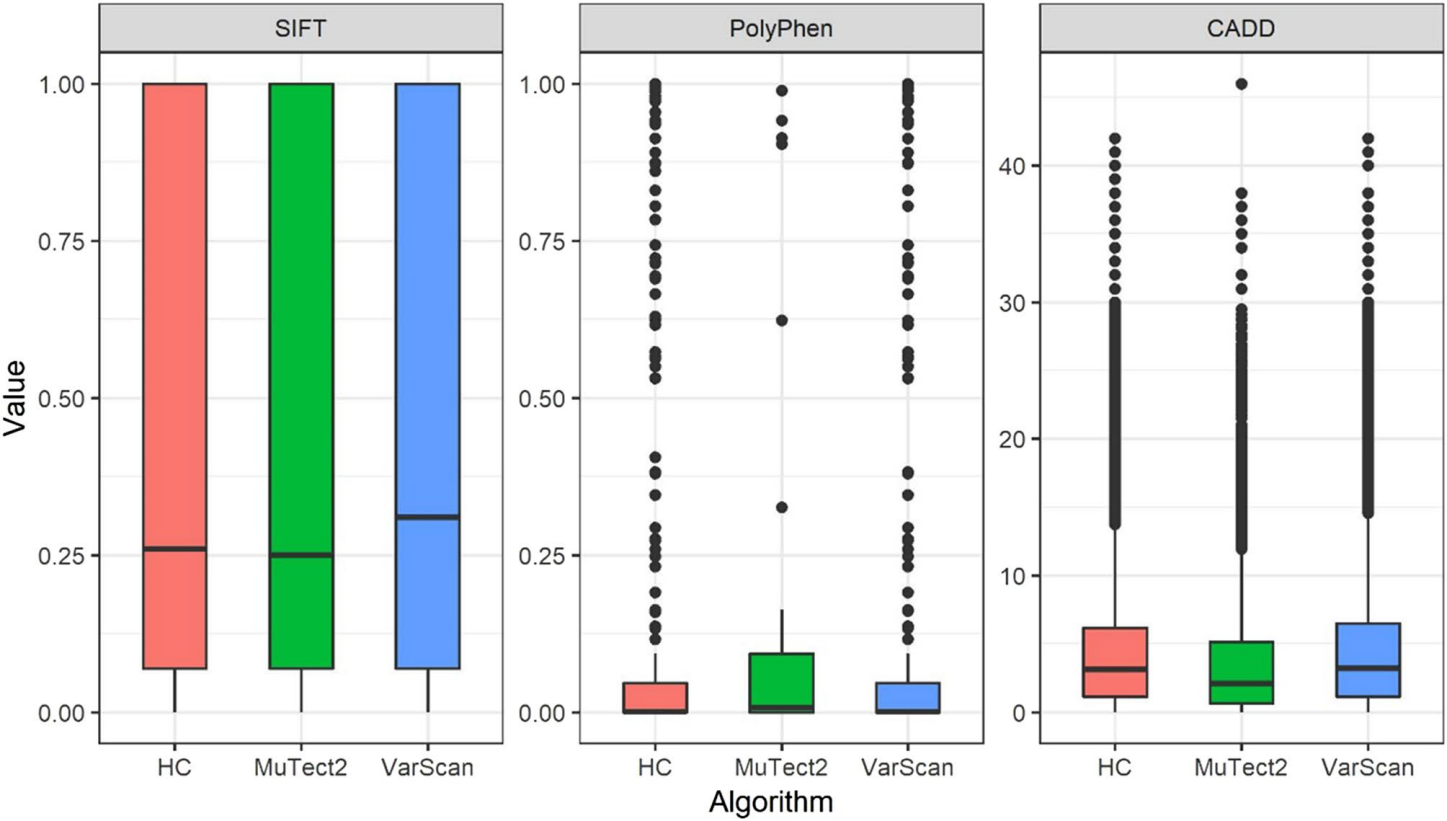
The distribution of deleteriousness scores of genetic variants called by three different variant callers represented by boxplots.

To infer the importance of genetic variants, we annotated the deleterious values of the SIFT, PolyPhen, and CADD algorithms that predict the intolerance of the variant by the conservation between species. For variants called using GATK HC, MuTect2 and VarScan, 2224, 1960, and 40 variants were annotated with SIFT, 2345, 2078, and 41 with PolyPhen, and 435,999, 307,152, and 16,719 with CADD, respectively (Fig. 2). Among the variants annotated using SIFT, 363 (16.32%), 342 (17.45%), and 9 (22.50%) deleterious variants were observed with scores < 0.05 for GATK HC, VarScan, and MuTect2, respectively. Of the variants with annotated PolyPhen scores, the numbers of deleterious variants with scores > 0.95 were 120 (5.12%), 109 (5.25%), and 1 (2.44%) for GATK HC, VarScan, and MuTect2, respectively. Among the variants annotated using CADD, the numbers of variants with scores > 15 were 16,364 (3.75%), 13,391 (4.36%), and 419 (2.51%) for GATK HC, VarScan, and MuTect2, and 7355 (1.69%), 6281 (2.04%), and 199 (1.19%) for deleterious variants with scores > 20, respectively.

### ClinVar for clinical significance

Table 2 shows the ClinVar annotations for clinical significance in compliance with the variant-calling algorithms. The numbers of drug_response, likelypathogenic, pathogenic, protective, and risk_factor mutations, which are clinically important, were 1504 (3.07%), 134 (0.27%), 405 (0.83%), 306 (0.62%), and 753 (1.54%) for GATK HC; 1354 (3.21%), 129 (0.31%), 364 (0.86%), 285 (0.68%), and 674 (1.60%) for VarScan; and 19 (1.08%), 16 (0.91%), 21 (1.19%), 7 (0.40%), and 10 (0.57%) for MuTect2, respectively. The average number of ClinVar significant variants of the patients was 769.43, the variants detected by only one caller were 16.5%, and those detected by two callers were 82.18%.

**Table 2:** The distribution by the ClinVar category of genetic variants according to three different variant callers.

Clinical significance	GATK HC	VarScan	MuTect2
Association	70 (0.14%)	67 (0.16%)	0 (0.00%)
Benign	31,816 (64.96%)	27,175 (64.50%)	1079 (61.20%)
Drug_response	1504 (3.07%)	1354 (3.21%)	19 (1.08%)
Likely_benign	8697 (17.76%)	7658 (18.18%)	404 (22.92%)
Likely_pathogenic	134 (0.27%)	129 (0.31%)	16 (0.91%)
Not_provided	3534 (7.22%)	2860 (6.79%)	78 (4.42%)
Other	276 (0.56%)	258 (0.61%)	5 (0.28%)
Pathogenic	405 (0.83%)	364 (0.86%)	21 (1.19%)
Protective	306 (0.62%)	285 (0.68%)	7 (0.40%)
Risk_factor	753 (1.54%)	674 (1.60%)	10 (0.57%)
Uncertain_significance	1483 (3.03%)	1305 (3.10%)	124 (7.03%)
Sum	48,978 (100.00%)	42,129 (100.00%)	1763 (100.00%)

**Figure 3:**
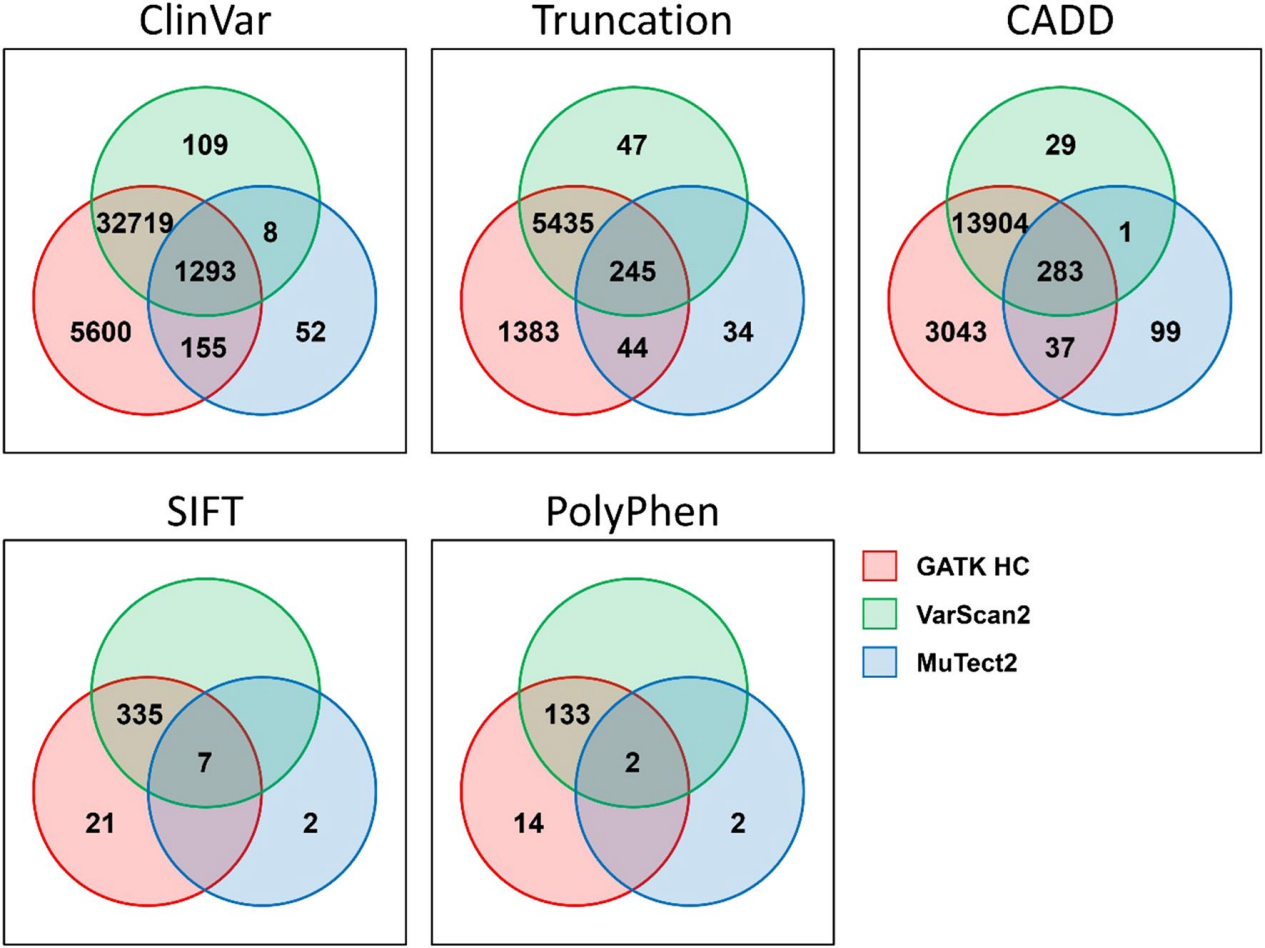
Summary of significant variants differently called by variant callers. (**a**) ClinVar annotated variants. (**b**) The consequences of truncation mutation. (**c**) Variants with deleterious sift scores < 0.05. (**d**) Variants with deleterious PolyPhen-2 scores > 0.85. (**e**) Variants with deleterious CADD scores > 15.

To visualize the distribution of differentially detected clinically significant variants, individual distributions of patients with mutations are presented in a Venn diagram. In Fig. 3, ClinVar is based on variants corresponding to drug_response, likely_pathogenic, pathogenic, protective, and risk_factor. Truncation is based on variations whose consequence is the loss of function. The SIFT score was 0.05 or less, the PolyPhen score was 0.85 or more, and the CADD score was 15 or more.

To characterize the differently called variants, we reviewed variants that included significant consequences, deleteriousness scores, and ClinVar annotations that GATK HC found but VarScan did not (Table 3). ABCA4 is an ATP-binding cassette (ABC) transporter (OMIM 601691; GenBank U88667). Diseases associated with ABCA4 include age-related macular degeneration and Stargardt disease^13,14^. Diseases associated with DHCR7 include Smith-Lemli-Opitz Syndrome and holoprosencephaly. There is much evidence associating the variant rs11555217 with disease^15,16^. Diseases associated with CYP4V2 include Bietti crystalline corneoretinal dystrophy and telangiectatic osteogenic sarcoma^17^. Diseases associated with CFTR include cystic fibrosis and Vas Deferens congenital bilateral aplasia^18^. This gene is a target of FDA-approved drugs and is known to be associated with ivacaftor, glyburide, bumetanide, crofelemer, and lumacaftor drugs^19–23^.

**Table 3:** The annotation information for clinically important variants that GATK HC found, but VarScan and MuTect2 were never found.

Symbol	Existing_variation	Consequence	SIFT	PolyPhen	CADD	ClinVar annotations
ABCA4	rs61750130	Missense_variant	0	0.716	28.1	Pathogenic, risk factor
ABCA4	rs140482171	Missense_variant	0.16	0.013	21.7	Likely pathogenic
DHCR7	rs11555217	Stop_gained			36	Pathogenic
ABCA4	rs1801581	Missense_variant	0.01	0.163	22.7	Pathogenic, risk factor
CYP4V2	rs199476189	Stop_gained	<NA>	<NA>	42	Pathogenic
CFTR	rs121909021	Missense_variant	0.02	0.531	27.5	Pathogenic
CFTR	rs78655421	Missense_variant	0	1	24.9	Pathogenic, drug response

## Discussion

With advances in NGS technologies in the past several years, genome or exome sequencing is now practiced in medicine. However, different NGS pipelines among institutions produce different variant calling results despite the same raw sequencing data, causing serious problems in clinical decision-making and genetic variant sharing. Variant calling, which is the result of diagnostic genetic tests, should be reproducible or replicable for use as a basis for clinical decision-making^12^. In breast cancer, various genomic factors, such as EGFR, BRCA1/2, ESR1, PIK3CA, and TP53, greatly influence clinical decisions^24^. However, if this information is not reproducible and replicable among medical institutions, it can cause confusion when making clinical decisions. The development of numerous NGS sequencing technologies, such as Illumina and BGI, has caused data-specific effects, making it difficult to build a uniform pipeline^8,9^. Therefore, we suggest that the entire pipeline throughout the variant-calling process, including raw sequencing data, should be shared to enhance the reproducibility of the genetic variants. All processing included in the NGS pipeline, such as the version of the programs, options, and additional files with each version, should be shared to reproduce or replicate the same genetic variant from the raw sequence. Of the genetic variants called by different NGS pipelines, we quantified how many important variants were missed, affecting clinical decision-making. As a result, we found that important variants affecting clinical decisions are found quite differently according to the variant-calling algorithm.

Several studies suggest that the result of variant calling differs by NGS preprocessing and variant-calling pipeline^25,26^. Moreover, the result of variant calling is different for different sequencers, despite using the same raw sequence data and NGS pipeline. Nevertheless, establishing a guideline with a uniform NGS pipeline for a single best practice is difficult because the performance of NGS pipelines differs by sequencer, purpose of the sequencing, and characteristics of the sample^27^. Therefore, there is the risk of making a clinical decision with a genetic variant in an institution that does not perform NGS pipeline because the institution cannot reproduce the result of the variant calling. Hence, details of the NGS pipeline for the entire variant-calling process are essential.

To evaluate the significance of the variants called by three different variant caller algorithms, GATK HaplotypeCaller, MuTect2, and VarScan, we used the consequence, deleteriousness score, and ClinVar classification. Consequences of variants, referred to as loss-of-function mutations, can be divided into truncation and non-truncation mutations. Truncation mutations have a profound impact on the loss of gene function. SIFT, PolyPhen, and CADD scores are algorithms that measure deleteriousness of genes based on conservation and protein structure. ClinVar annotated variants are clinically significant genetic variants categorized into pathogenic, drug response, risk factor, and more, which are important information in making clinical decisions. Truncation mutations, deleterious variants, and clinically significant variants have different results depending on the variant-calling algorithm, even though they are variants that have a large effect on gene function (Fig. 3). Thus, NGS pipelines that produce different variant calling results can have a significant impact on clinical decisions based on genetic variants.

Our study has some limitations. We only measured variant differences based on variant callers. From the read alignment algorithm to the final variant-calling process within the entire NGS pipeline, various factors can affect variant calling. We could not test all of them due to the combination explosion, but we focused on variant calling. A replication study of the genetic testing pipeline used in hospitals is needed. From the NGS pipeline information used in hospitals, we need to test whether the variant calling results can be reproduced from the same raw sequence data.

In conclusion, our results show that clinically important variants are differently called by variant callers, thus affecting clinical decisions. This means that variant calling outcomes are not reproducible without detailed NGS pipeline information. Therefore, we suggest that the pipeline throughout the variant-calling process, including raw sequencing data, should be shared for effective genetic variant sharing and clinical decision-making.

## Methods

### Raw sequencing samples

Raw sequence files from massive parallel sequencing of blood DNA from 105 breast cancer patients were downloaded from the NCBI Sequencing Read Archive (SRA) database (SRP174001). These targeted data were sequenced for the coding and regulatory regions of 509 genes selected from PharmGKB and Phenopedia, where a number of important variants are located for clinical decisions^2,28^. SRA files were downloaded using 'prepetch' version 2.9.4 of the SRA Toolkit (https://www.ncbi.nlm.nih.gov/sra/docs/toolkitsoft/). The SRA files were converted to paired sequence FASTQ format files using fastq-dump of the SRA Toolkit (https://ncbi.github.io/sra-tools/fastq-dump.html). Quality assessment of the paired sequence reads was performed using FastQC version 0.11.8, followed by adaptor removal and read trimming (http://www.bioinformatics.babraham.ac.uk/projects/fastqc/)^29^.

### Pre-processing of DNA resequencing data

The raw FASTQ files and paired sequence data were aligned to the human genome hg38 assembly using the Burrows-Wheeler Aligner, BWA program, version 0.7.12, and were transformed into a sequence alignment map (SAM) format^30^. Using SAMtools version 1.9, sequence data in SAM format was compressed into Binary Alignment Map (BAM) format by view command, and the aligned sequence reads were sorted with leftmost coordinates by sort command. Read groups are added to aligned sequence files using the’ AddOrReplaceReadGroups’ module in Picard. Next, SAMtools was used to prepare index referencing and BAM files^31^. After preparing these files, GATK version 3.8 was used to perform Realigner Target Creator and Indel Realigner to locally realign regions containing insertions and deletions to correct misaligned reads^6^. Base quality scores were adjusted using GATK BaseRecalibrator with the dbSNP build 138 and 1000-genome gold standard indels provided by the GATK Resource bundle standard files for working with human resequencing data (https://software.broadinstitute.org/gatk/download/bundle)^32,33^. The sequencing target section was extracted using the bedtools intersect version 2.26 with indexing^34^. Finally, Picard MarkDuplicates v1.93 was used to identify duplications with the option to flag and remove duplicate reads.

### Small variant detection

After preprocessing the DNA sequencing data, we detected single nucleotide variants (SNVs) using three algorithms. VarScan and GATK HaplotypeCaller (HC) were used to find genetic variants between the sample DNA sequence compared with the reference sequence^35^. Somatic variants were called using GATK MuTect2^36^. Variants called by a mixture of germline and somatic variant calling tools were compared based on the assumption that NGS pipeline information was not properly shared during the communication process for the genetic variant of the patient. Reference genome databases, dbSNP build 138, and COSMIC, a source of commonly mutated genes, were used for the variant-calling argument.

### Genetic variant annotation

The Ensembl Variant Effect Predictor (VEP) was used to determine the effect of genetic variants derived from the three variant callers, HC, MuTect2, and VarScan^37^. The mutation consequence, SIFT score, PolyPhen score, CADD score, and ClinVar annotations were determined to examine the effect of differently called variants on variant callers^3,38–40^. Consequences were divided into truncating and non-truncating mutations. While truncating mutations included nonsense mutations, frameshift deletions, frame shift insertions, and splice-site mutations, non-truncating mutations included missense mutations, in-frame deletions, in-frame insertions, and nonstop mutations. To evaluate the significance of genetic variant effects, SIFT, PolyPhen, and CADD algorithms for predicting the deleteriousness of variants were used. SIFT score < 0.05, PolyPhen > 0.95, and CADD > 15 were defined as deleterious variants. The clinical significance of genetic variants was cataloged by making comparisons in ClinVar (http://www.ncbi.nlm.nih.gov/ClinVar/).
